# Technology-aided assessments of sensorimotor function: current use, barriers and future directions in the view of different stakeholders

**DOI:** 10.1186/s12984-019-0519-7

**Published:** 2019-04-29

**Authors:** Camila Shirota, Sivakumar Balasubramanian, Alejandro Melendez-Calderon

**Affiliations:** 1 Department of Health Sciences and Technology, ETH Zurich, Zürich, Switzerland; 2 Department of Bioengineering, Christian Medical College, Bagayam, Vellore, Tamil Nadu 632002 India; 3 cereneo Advanced Rehabilitation Institute (CARINg), cereneo - Zentrum für Interdisziplinäre Forschung, Vitznau, Switzerland; 4 Department of Physical Medicine and Rehabilitation, Northwestern University, Chicago, USA

**Keywords:** Sensorimotor assessments, Neurorehabilitation technologies, Outcome measures, Rehabilitation, Physical therapy

## Abstract

**Background:**

There is growing interest in the use of technology in neurorehabilitation, from robotic to sensor-based devices. These technologies are believed to be excellent tools for quantitative assessment of sensorimotor ability, addressing the shortcomings of traditional clinical assessments. However, clinical adoption of technology-based assessments is very limited. To understand this apparent contradiction, we sought to gather the points-of-view of different stakeholders in the development and use of technology-aided sensorimotor assessments.

**Methods:**

A questionnaire regarding motivators, barriers, and the future of technology-aided assessments was prepared and disseminated online. To promote discussion, we present an initial analysis of the dataset; raw responses are provided to the community as Supplementary Material. Average responses within stakeholder groups were compared across groups. Additional questions about respondent’s demographics and professional practice were used to obtain a view of the current landscape of sensorimotor assessments and interactions between different stakeholders.

**Results:**

One hundred forty respondents from 23 countries completed the survey. Respondents were a mix of Clinicians (27%), Research Engineers (34%), Basic Scientists (15%), Medical Industry professionals (16%), Patients (2%) and Others (6%). Most respondents were experienced in rehabilitation within their professions (67% with > 5 years of experience), and had exposure to technology-aided assessments (97% of respondents). In general, stakeholders agreed on reasons for performing assessments, level of details required, current bottlenecks, and future directions. However, there were disagreements between and within stakeholders in aspects such as frequency of assessments, and important factors hindering adoption of technology-aided assessments, e.g., Clinicians’ top factor was cost, while Research Engineers indicated device-dependent factors and lack of standardization. Overall, lack of time, cost, lack of standardization and poor understanding/lack of interpretability were the major factors hindering the adoption of technology-aided assessments in clinical practice. Reimbursement and standardization of technology-aided assessments were rated as the top two activities to pursue in the coming years to promote the field of technology-aided sensorimotor assessments.

**Conclusions:**

There is an urgent need for standardization in technology-aided assessments. These efforts should be accompanied by quality cross-disciplinary activities, education and alignment of scientific language, to more effectively promote the clinical use of assessment technologies.

**Trial registration:**

NA; see Declarations section.

**Electronic supplementary material:**

The online version of this article (10.1186/s12984-019-0519-7) contains supplementary material, which is available to authorized users.

## Background

Neurorehabilitation technologies are increasingly viewed as tools for quantifying and understanding sensorimotor problems, with potential beyond their use as therapeutic tools. It is commonly stated that technology can overcome some of the limitations of traditional clinical assessments by providing more objective, sensitive, reliable and time-efficient measurements [1–5]. However, despite the rich literature on technology-driven assessment of sensorimotor functions, the assessment tools currently used in clinics are generally limited to standard clinical scales and a trained clinician’s observation of a patient’s ability. It is unclear why neurorehabilitation technologies are not ubiquitous in routine clinical assessment of sensorimotor functions.

The *status quo* raises several questions about the advancement of sensorimotor assessments: what are the main uses and features of technological assessments? What hinders their further use, especially in the clinic? What do clinicians consider important to use these tools to aid in clinical decision-making? Answers to these questions can help technology developers create tools with meaningful translation to clinical practice.

In this context, there is need for a critical and open discussion among different stakeholders—with different points of view—about the current status and the future of the field. To this end, we conducted a workshop at RehabWeek 2017 in London (UK). This workshop had two components: (a) a survey prior to the workshop to gather the perceptions of different stakeholders, including patients, about the field; and (b) a multi-stakeholder panel discussion at RehabWeek with clinicians, engineers, researchers and technology providers. The aim of the workshop was to foster discussion about the role of technology in assessments by bringing together people from different backgrounds. These activities aimed at (i) taking a critical look at current approaches to sensorimotor assessments in neurorehabilitation; and (ii) trying to reach an integrated view of potential avenues for research that can lead to clinically meaningful and translatable results. Here, we report an initial analysis of the survey responses to initiate a deeper discussion regarding this topic; we further provide the raw responses (Additional file 1 - Responses) for future detailed analyses.

## Methods

### Online questionnaire

The survey was conducted as an online questionnaire designed to gather the views of different stakeholders in sensorimotor assessments for neurorehabilitation. The items of the questionnaire probed the perceived current state of assessments, along with the appropriate future directions for the field. The questionnaire had both common and stakeholder-/profession-specific questions (Table 1) and was divided into three sections. Section I had questions on the respondent demographics and profession. The latter was used to direct respondents to profession-specific questions in Section II. Section III consisted of common questions focused on the current views, barriers, and future of technology-aided assessments in neurorehabilitation. The questions were based on Duncan and Murray [6], who categorized the barriers for performing routine clinical assessments into: (i) Knowledge, Education, and Perceived Value; (ii) Support/Priority; (iii) Practical Considerations; and (iv) Patient Considerations.

**Table 1:** Structure of the online questionnaire. Refer to Additional file 1 for the specific questions (indicated by Qx)

Section	Target professional background	Topics covered
Section I Demographics	All	• Gender (Q1) • Location (Q2) • Categorization: Patient/Non-patient (Q3) • Years of experience in rehabilitation (Q4) • Exposure to technology-aided assessments (Q5) • Profession (Q6)
Section II ^1^ Profession-specific questions	Clinicians	• Work environment & experience with technology (Q7-Q8) • Frequency of assessments with and without technology (Q9-Q11) • Interaction with engineers (Q12)
	Engineers (research or medical industry)	• Familiarity with different technologies (Q13) • Interaction with clinicians and patients (Q14-Q15) • Perceptions about routine assessments (Q16)
	Neuroscientists	• Interaction with clinicians and patients (Q24-Q25) • Perceptions about routine assessments (Q26-Q26) • Perceptions about using neuroscience paradigms as clinical assessments (Q27-Q28)
	Hospital/clinic administrators	• Use of technology for rehabilitation and assessments (Q29-Q30) • Frequency of assessments in their institution (Q31)
	Medical Industry (non-engineering positions)	• Type of technology provided (Q32) • Market for technology-based assessments (Q33; Q35-Q36) • Effort put into technology-based assessments (Q34)
	Patients	• Rehabilitation environment (Q17) • Frequency of assessments with and without technology (Q18-Q19) • Perceived value of assessments (Q20) • Knowledge of results (Q21) • Types of technologies used (Q22-Q23)
Section III Motivators, barriers and opinions on the future of technology-aided assessments	All	• Duration of ideal assessment (Q37) • Motivators for routine assessments with and without technology (Q38) • Level of detail required for different assessment purposes (Q39) • Major bottlenecks in technology-based assessments (Q40-Q41) • Focus for the next 5 years to promote technology-based assessments (Q42) • Open questions/comments (Q43-Q45)

^1^Respondents that selected Policy maker, Insurance representative, or Other (free text) as their profession did not complete Section II

The online questionnaire was elaborated in Google Forms and was pilot tested (to verify content and clarity) with therapists and human movement scientists from cereneo Center for Neurology and Rehabilitation (Switzerland), the University Hospital Zurich (Switzerland), and rehabilitation engineers from ETH Zurich (Switzerland). The final version of the questionnaire was available online from 12 June to 08 August 2017. The link to the questionnaire was advertised in: rehabrobotics listserv, RehabWeek newsletter, RehabWeek Facebook and Twitter pages, cereneo AG Facebook and Twitter pages, personal LinkedIn accounts of the authors, as well as through the personal network of the authors, workshop co-organizers and speakers through email. The complete online questionnaire is provided (Additional file 2 - Questionnaire).

### Analysis

The responses for each question were summarized according to the type of question:

1. Questions with only one selectable option (e.g., Q1),
2. Questions with multiple selectable options (e.g., Q5),
3. Questions with multiple items, with only one selectable option per item (e.g., Q8),
4. Questions with multiple items that need to be ranked (e.g., Q9), and
5. Open-ended questions with written responses. (e.g., Q44)

QT1- and QT2-type questions were summarized by counting the number of times each option was selected by the respondents. Responses for QT3-type questions were summarized by generating a 2D array of counts, where the rows corresponded to the different items in the question, and the columns corresponded to the different options to be selected. A similar analysis was first carried out for QT4-type questions, which created a 2D array summarizing the ranking of the different items in the question; row-wise weighted averages of the ranks were calculated for each item, where the weights were the corresponding rank values. QT5-type questions were summarized manually by grouping similar comments together. Reported percentages were rounded to the closest integer.

To understand the viewpoints from different stakeholders, respondents were prompted to select a single profession they most identified with. Based on their response, they were categorized into different groups (Table 2). Based on this categorization, the overall results for each question were the average results across the stakeholder groups.

**Table 2:** Categorization of professions in stakeholder groups for analysis

Stakeholder category	Selected profession (Q6)
Clinician	- Medical doctor - Therapist (clinic-focused) - Therapist (research-focused)
Research Engineer	- Engineer (academic or non-profit institution research)
Basic Scientist	- Neuroscientist - Other (free text) with the keyword (Mov* Scien* OR Clin* Scien*) AND NOT Indus* (e.g., movement scientist, clinical scientist)
Medical Industry	- Engineer (medical industry) - Manager (medical industry) - Sales representative of medical technologies - Other (medical industry) - Other (free text) with the keyword Indus* (e.g., therapist in industry)
Hospital Administrator	- Hospital / clinic administrator
Policy Maker	- Policy maker (e.g., work for a government organization) - Insurance representative
Patient	Receiving therapy due to a neuromuscular disorder (Q3).
Other	None of the above

## Results

### Respondent background and experience

**Fig. 1:**
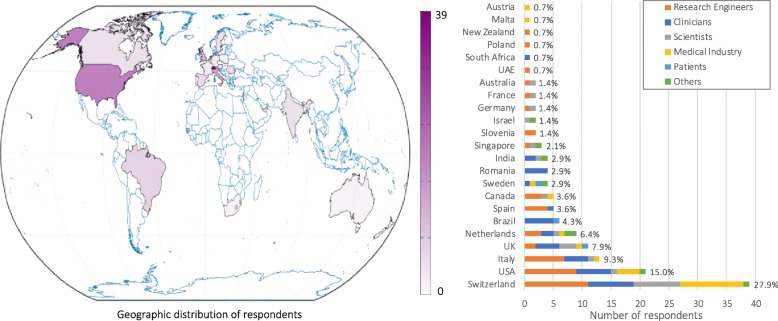
Geographic distribution of questionnaire respondents. Countries are shaded according to the number of respondents; the list of countries shows their relative representation. [Q2: What country do you live/work in?]

A total of 140 responses were obtained (52% male, 47% female, 1% preferred not to say; Q1). Responses were from 23 different countries, mainly in Europe and North America (Q2; Fig. 1). Thirty-seven percent of respondents declared that they would be attending RehabWeek (Q43).

**Fig. 2:**
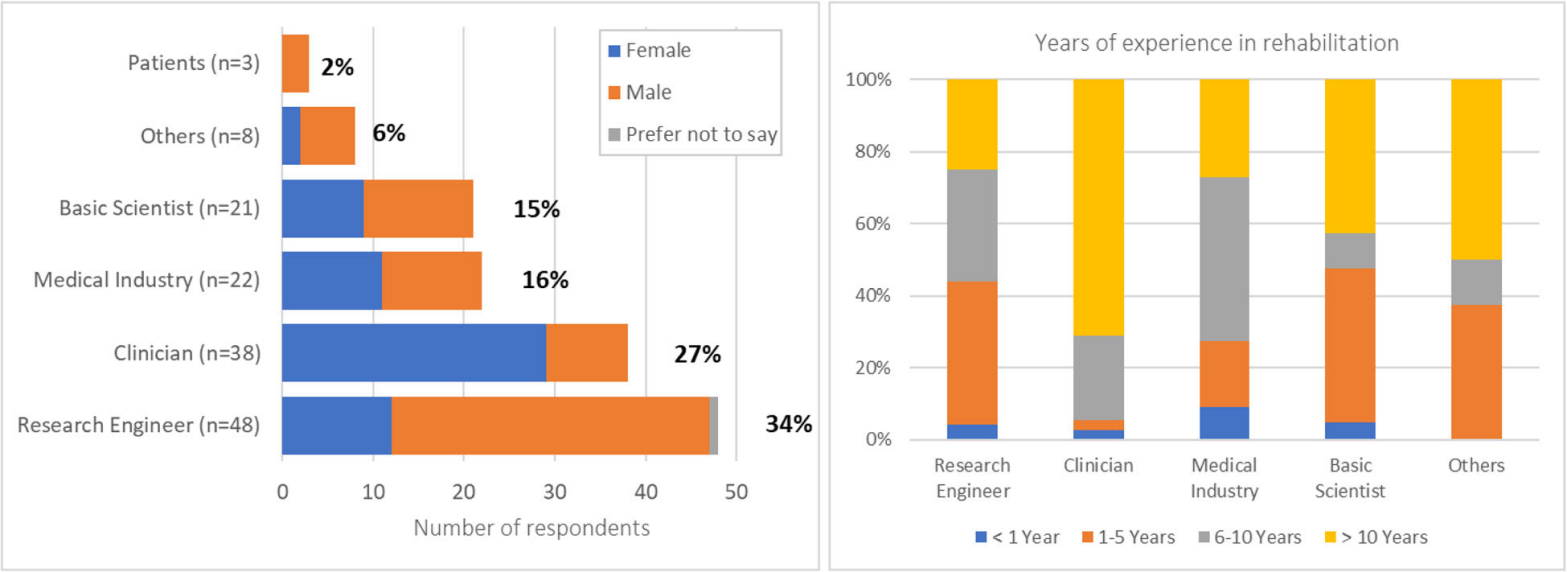
Respondent demographics per stakeholder group (left) [Q1: What is your gender?; Q6: What is your profession? (according to Table 2)] and years of experience in rehabilitation (right) [Q4: How many years of professional experience do you have in the field of rehabilitation? (Including: clinical work, research, technology development, sales, policy making, etc.)]

Most respondents were Research Engineers (34%), followed by Clinicians (27%), Medical Industry professionals (including Managers, Sales representatives, Engineers; 16%), Basic Scientists (including Neuroscientists, Human Movement and Clinical Scientists; 15%), Other professionals (6%), and Patients (2%) (Q6; Fig. 2, left). We did not have responses from Hospital/Clinic Administrators, Policy Makers or Insurance Representatives; thus, questions related to these stakeholders were not included in this manuscript.

Forty-two percent of respondents (not including Patients) had more than 10 years of experience in their respective fields (Q4; Fig. 2, right); Clinicians had the most experience, while respondents from other professional backgrounds were more evenly split between junior (up to 5 years of experience) and senior (more than 5 years of experience). Clinicians worked (Q17) mostly in Inpatient units (34% in small and 21% in large clinics/hospitals), followed by Outpatient units (18% in small and 11% in large clinic/hospital), Home care (5%), Skilled nursing facilities (3%) and Others (8%). From the three patients, one was treated in an acute-care unit, one in a small inpatient unit, and one in a large outpatient unit (Q17).

#### Interdisciplinary interactions

In general, most respondents had some interdisciplinary interactions with other professional groups. About 66% of Clinicians had previously interacted with technology developers on at least one case, with 24% (overall) having done so on more than 5 separate occasions (Q12; Table 3). Among the Research Engineers, 90% had interacted with patients and therapists, with 52% (overall) having interacted with more than 10 patients (Q14) and 21% (overall) with more than 10 therapists (Q15). Among the Medical Industry (Engineers), all had directly interacted with patients, with 50% (overall) having interacted with more than 10 patients (Q14) and 10 therapists (Q15). Among Basic Scientists (Neuroscientists), 94% had directly interacted with patients and 82% with therapists; 76% (overall) reported interactions with more than 10 patients (Q24) and 41% (overall) reported interactions with more than 10 therapists (Q25).

**Table 3:**
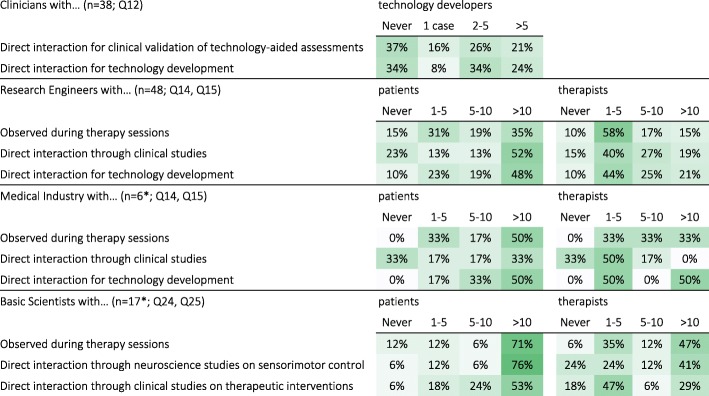
Interdisciplinary interactions between Clinicians, Engineers, Basic Scientists and Patients. [Q2, Q14, Q15, Q24, Q25: In your work, what kind of interactions have you had with: i) technology developers (Q12), ii) patients (Q14 & Q24) and iii) therapists (Q15 & Q25)?]

#### Use of technology at work

**Fig. 3:**
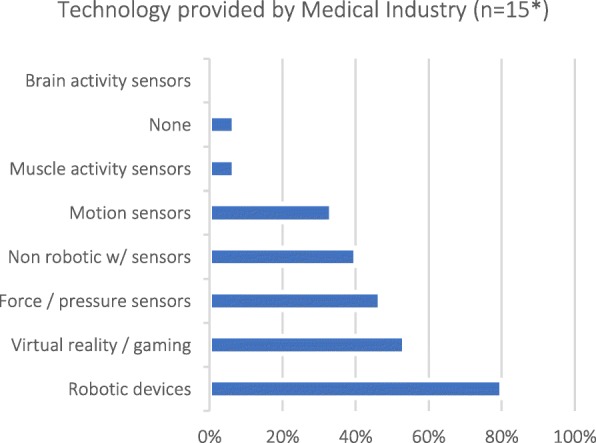
Types of technology provided by Medical Industry [Q32: What kind of technologies do you provide?]; note that selecting more than one category was possible. *Only respondents that selected “Manager (medical industry)”, “Sales representative of medical technologies” or “Other (medical industry)” as their profession (Q6) were asked this question

To gauge respondents’ proficiency with technology, rather than focus on potential age-related barriers, we asked about their exposure to different types of technological devices at work. Eighty percent of Medical Industry (non-Engineer) respondents were providers of robotic devices (Q32; Fig. 3).

**Fig. 4:**
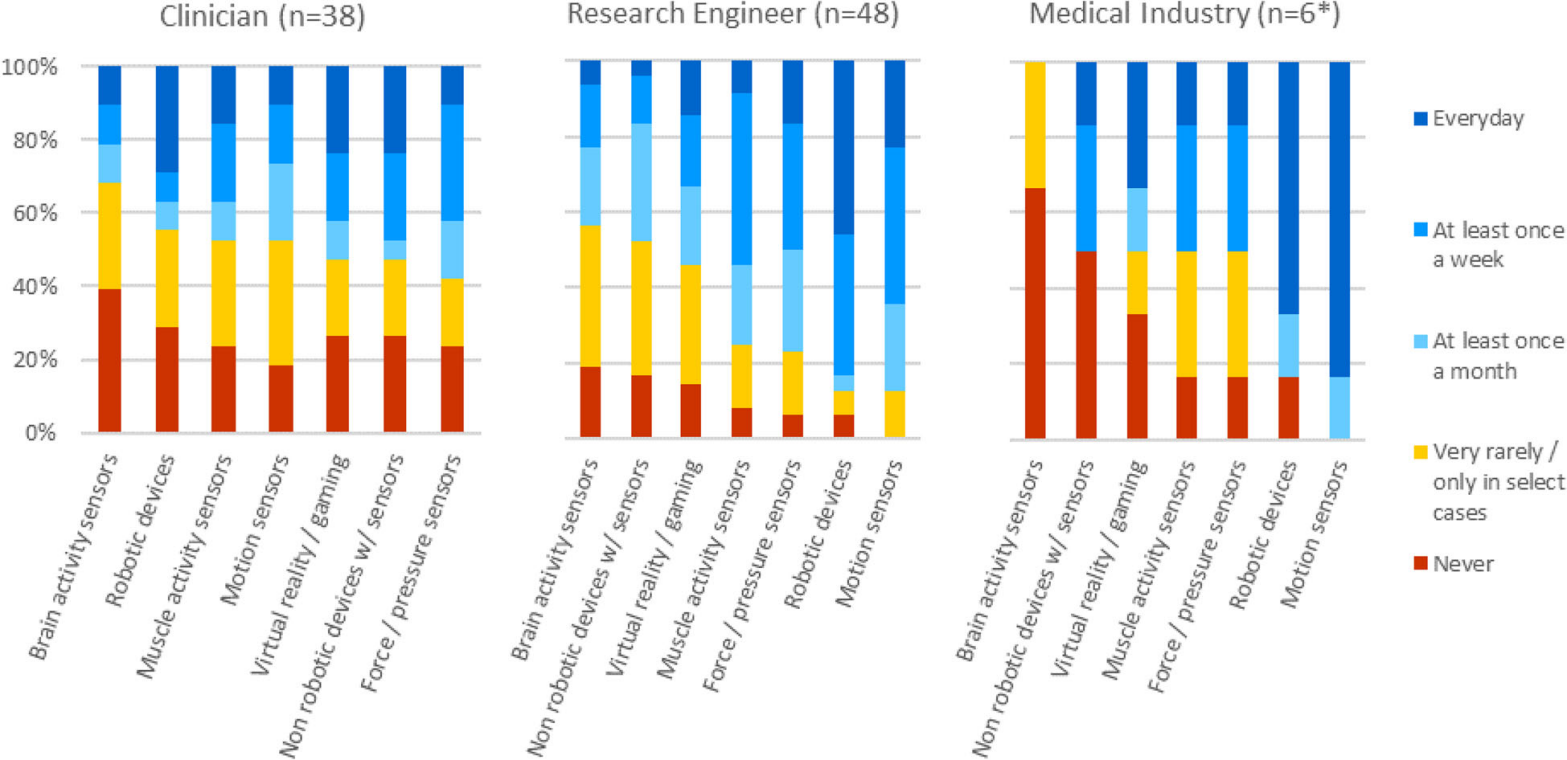
Use of technology at work by different stakeholders. [Clinicians: Q8: How often are these technologies used in your work (not restricted to assessments)?; Engineers (research and medical industry): Q13: How often are these technologies used in your work environment?]. *Only Medical Industry respondents that selected “Engineers (medical industry)” as their profession (Q6) were asked this question

About 75% of Clinicians worked in environments where technology (not exclusively assessment-related) is used routinely, with 50% (overall) working with technology on a regular basis (Q8; Fig. 4). Of the listed technology types, most clinicians had been exposed to motion sensors, and least with brain activity-related technologies. In contrast, the majority of Engineers (research and industry) used technology on a regular basis (Q13; Fig. 4), especially robots and motion sensors.

#### Exposure to technology-aided assessments

**Fig. 5:**
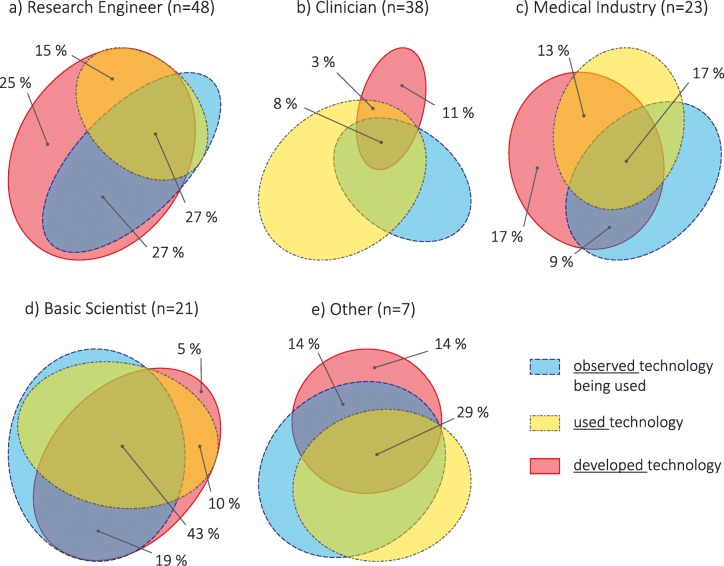
Exposure to technology-aided assessments – observed (blue, dashed outline), used (yellow, dash-dotted outline), and developed (red, solid outline) – per stakeholder group: **a** Research Engineer, **b** Clinician (two participants stated ‘no experience’), **c** Medical Industry (two participants stated ‘no experience’), **d** Basic Scientist, **e** Other. Areas proportional to counts within group; adapted from diagrams generated with [7]. We show the percentages associated to respondents who have developed technology (i.e. developed, developed AND observed, developed AND used, developed AND observed AND used) [Q5: What kind of exposure have you had with technology-aided assessments?]

Almost all respondents had exposure to technology-aided assessments, through observation, use or development, with only 5% of Clinicians and 5% of Medical Industry reporting no experience (Q5; Fig. 5). All stakeholder groups had individuals who had developed technology: 94% of Research Engineers, 21% of Clinicians, 59% of Medical Industry professionals, 59% of Basic Scientists, and 50% of Others. Surprisingly, a large percentage of people who had developed had not observed technology being used: 43% of Research Engineers, 54% of Medical Industry professionals, 63% of Clinicians, 29% of Basic Scientists, and 25% of Others; this included people with senior level experience in almost all groups. Basic Scientists presented the most overlap between exposure types, suggesting collaboration and interaction between developer and user groups.

### Current state of assessments

#### Frequency of standard assessments in the clinic

Research Engineers, Basic Scientists and Medical Industry professionals underestimated how often Clinicians perform assessments. Approximately 75% of Clinicians reported using standardized clinical assessments on a regular basis (at least once a month; Q10), while technology-based assessments were used less often (Q11). In contrast, half or more of Research Engineers (Q16), Medical Industry professionals (Q16) and Basic Scientists (Q26) believed that, for each patient, sensorimotor assessments are performed only at entry and discharge (Table 4), although many believe that assessments are performed on a regular basis (at least once a month; 34%, 50%, and 30%, respectively). Patient responses (Q18-Q19) generally followed Clinician responses.

**Table 4:**
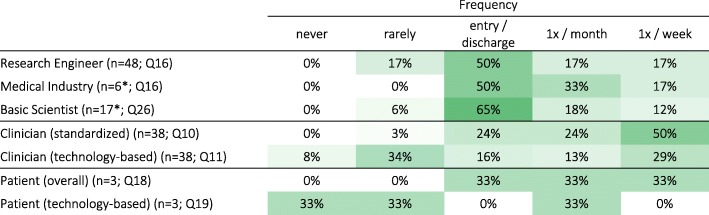
Frequency of sensorimotor assessments as predicted by Research Engineers, Medical Industry and Basic Scientists (yellow), as practiced by Clinicians (green), and as experienced by Patients (blue; results in percent respondents per group). Darker shades indicate higher percentages. [Q16, Q26: How often do you think therapists perform standard clinical assessments on a given patient during routine clinical practice? | Q10: How often do you (or your personnel) perform standard clinical assessments on a given patient during routine clinical practice? | Q11: How often do you (or your personnel) use any kind of technology to perform assessments on a given patient during routine clinical practice? | Q18: How often do you do clinical assessments during your therapy sessions | Q19: How often do your therapists use technological tools to assess you during therapy sessions?]

All patients reported that they are made aware of the results from their assessments (Q21). Two out of three Patients reported that they see value in having their abilities assessed by a professional (Q20), while one said ‘maybe’. None of the patients in this study use technological tools to assess their own progress (Q22-Q23).

#### Preferred sensorimotor assessment tools

In their practice, Clinicians reported a strong preference for the use of standard clinical assessments (scales or questionnaires) and showed least preference for non-standardized tasks or questionnaires (Q9; Table 5). Low-tech and technology-based measurements obtained similar weighted rank (3.63 out of 7), but their response distributions were different, with many respondents showing a strong dislike for technology-based measurements (47% had preference below or equal to 3 out of 7, where 1 is the least and 7 is the most preferred method). It is unclear how constrained responses to this question were to internal standards or requirements of the institutions where Clinicians work.

**Table 5:**
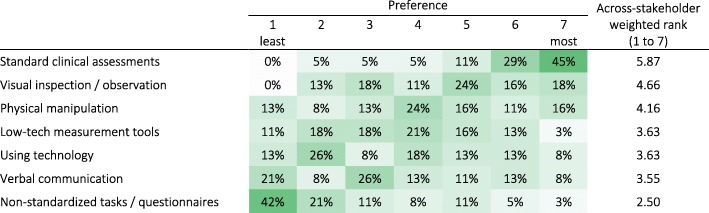
Clinician preference for different sensorimotor assessment methods (in percent Clinician respondents; n = 38). Darker shades indicate higher percentages. [Q9: How do you assess the sensorimotor ability of your patients?]

#### Reasons for doing assessments

Stakeholders generally agreed that the main motivator for carrying out assessments was for “individual” reasons (*n* = 140; across-stakeholder weighted rank of 2.6 on a scale from 1 to 3), i.e., for treatment planning and evaluation for each specific patient (Q38; Table 6). Medical Industry professionals and Patients also scored institutional reasons, i.e., healthcare delivery planning and evaluation, highly (average of 2.1 and 2.3, respectively).

**Table 6:**
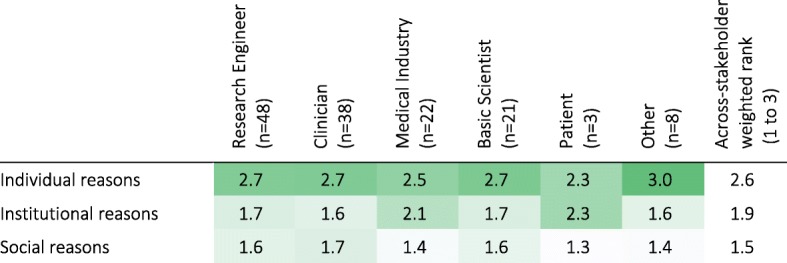
Motivators to perform and document assessments (1–Least Important, 3–Most Important). Darker shades indicate higher percentages. [Q38: What are the main motivators to perform and document results from routine assessments (standard clinical or technology-aided)?]

There was general agreement among the stakeholders about the reasons for performing assessments, and the level of detail required for different purposes. The level of detail required for assessments varied based on the objective of the assessment (Q39; Table 7). Assessments performed to determine invasive interventions were perceived to require the most detail (across-stakeholder weighted rank of 3.3 on a scale from 1 to 4), while monitoring progress at home required the least (across-stakeholder weighted rank of 1.6). Therapy planning and evaluation of different therapeutic techniques required more detail than for reporting progress to government bodies/insurance companies. Discussions among clinicians were reported to require more detail than when discussing progress with patients. Interestingly, the three Patients that participated in the survey were not interested in details to monitor their own progress; they also considered that discussing patient progress required more detail than evaluating/determining interventions, which disagrees generally with all other stakeholders. Further, Clinicians and Basic Scientists tended to think more detail is needed to determine therapy interventions than when gathering evidence for effectiveness of interventions, while Medical Industry professionals and Others tended to think the opposite; Engineers considered a similar level of detail is needed for both objectives.

**Table 7:**
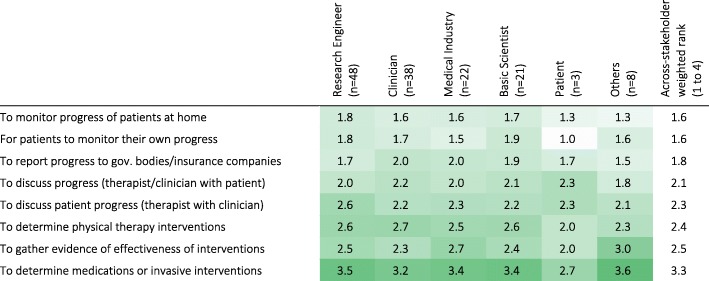
Minimum level of detail required in sensorimotor assessments for different objectives according to stakeholder groups (weighted rank per group). 1 – indicates activity-level assessments (low level of detail), while 4 – indicates assessment at the structural- and biological-levels (high level of detail). Darker shades indicate higher percentages. [Q39: In your opinion, which is the minimum level of detail needed in the quantification of sensorimotor problems for the following objectives?]

#### Current effort in industry for technology-based assessments

Medical Industry (non-Engineer) professionals reported that sensorimotor assessments are a selling point for devices (*n* = 15; average of 3.5 on a scale from 1 to 5; Q35), with most interest shown by researchers, followed by therapists, medical doctors, insurance companies, patients, hospital administrators and regulatory bodies (Q33; Table 8). Three out of fifteen respondents from Medical Industry reported that they only sell assessment devices; the rest reported investing the same amount of resources in sensorimotor assessment features as in therapeutic features of their devices (*n* = 12; average of 2.1 on a scale from 0 to 5, where 0 - Zero resources, 2 - Same, 4 - Double, and 5 - More than Double; Q34).

**Table 8:**
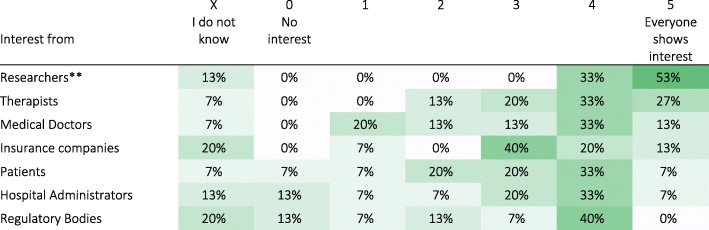
Interest in assessment tools by different groups as reported by Medical Industry (n = 15*). Darker shades indicate higher percentages. [Q33: What is the current interest in assessment tools?]

*Only Medical Industry (non-Engineers), i.e., respondents that selected entries different than “Engineer (medical industry)” as their profession (Q6), were asked this question

**To avoid confusion, the term “Researchers” is used here in a general sense

### Future of technology-aided assessments

All professionals from Medical Industry (excluding Engineers) believe there will be an increase in market demand for assessment tools in the upcoming years (Q36). This is a positive sign that should motivate improvements and systematization of current tools and techniques in technology-aided sensorimotor assessments.

#### Duration of an ideal assessment protocol

Across all stakeholders, 6–15 min per patient per week was considered as the most reasonable amount of time for a sensorimotor assessment including setup time (Q37; Table 9), with the majority of respondents suggesting a time between 1 min and 30 min and none favoring assessments longer than 60 min. Medical Industry professionals tended to strongly prefer shorter assessment durations (1–5 min); Medical Industry professionals, Research Engineers and Patients had strong preferences for specific time intervals, with half or more of each group selecting one time interval.

**Table 9:**
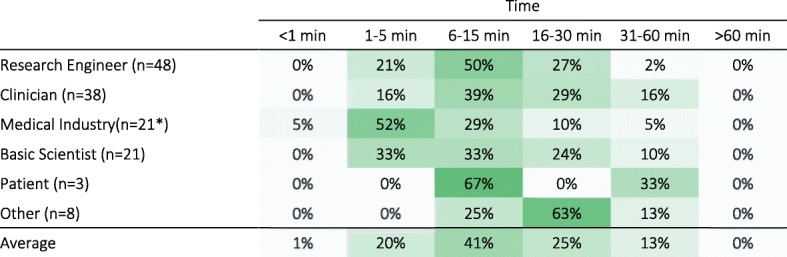
Preferred amount of time for sensorimotor assessments per patient per week (in percent respondents per group). Darker shades indicate higher percentages. [Q37: What is a reasonable amount of time for a sensorimotor assessment that has to be performed at least once a week (per patient) - from beginning of setup to end of undoing setup?]

*one participant did not answer this question

#### Bottlenecks in current assessments

Across all stakeholders, practical considerations were ranked as the most hindering factor to the use of standard and technology-aided sensorimotor assessments in the clinic, followed by knowledge/education/perceived value, support/priority and finally patient considerations (Q40; Table 10). This ranking generally agreed with the responses from all stakeholder groups, except Patients and Others. Of note, knowledge/education/perceived value was scored highly by Clinicians and Basic Scientists; support/priority by Patients; and patient considerations by Other professionals.

**Table 10:**
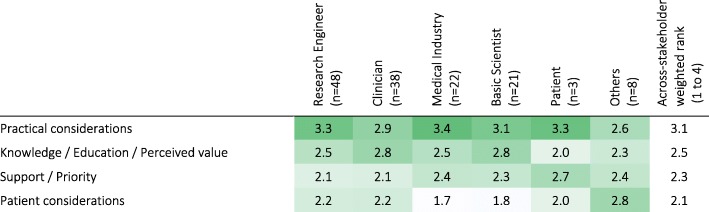
Importance of factors currently hindering the use of (standard and technology-aided) sensorimotor assessments in the clinical practice according to stakeholder groups (weighted rank per group). Darker shades indicate higher rank. [Q40: How much do issues in each of the following categories hinder the routine use of assessments (standard clinical or technology-aided) in the clinical practice?]

Specifically for technology-aided sensorimotor assessments, lack of time to perform assessments and the cost of technologies were considered the most important factors currently hindering their use (Q41; Table 11). The lack of reimbursement for assessment time and lack of educational programs on how to use technology were the least important factors. Interestingly, Medical Industry professionals and Research Engineers differed from the other stakeholder groups, respectively emphasizing lack of reimbursement, and lack of standardization and understanding how to interpret and use assessment results as most important.

**Table 11:**
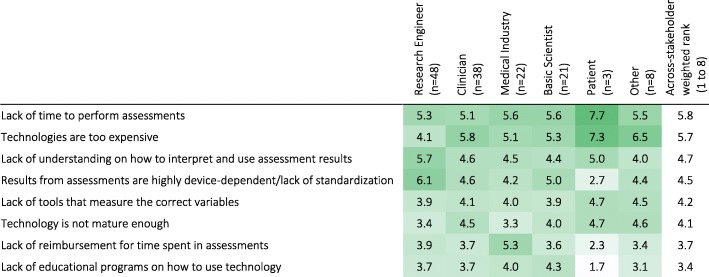
Importance of factors currently hindering the use of technology-aided sensorimotor assessments in the clinical practice according to stakeholder groups (weighted rank per group; rank: 1 = least important, 8 = most important). Darker shades indicate higher percentages. [Q41: What are the major bottlenecks in implementing technology-aided sensorimotor assessments in routine clinical practice?]

#### Plan for the next 5 years

To advance the field in the near future, standardization of technology-aided assessments, better understanding of the link between different levels of assessment (i.e., activity, high-level function, low-level function, and structural/biological), and reimbursement/economic feasibility were considered the most important activities to focus on in the coming years (Q42; Table 12). Cross-disciplinary education/communication and definition of terms and taxonomies were considered less important.

**Table 12:**
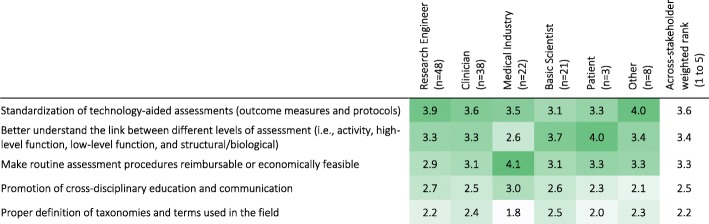
Importance of types of activities to do in the next 5 years to facilitate the use of new technologies and methodologies in the clinical practice according to stakeholder group (weighted rank per group). 1: lowest priority; 5: highest priority. Darker shades indicate higher percentages. [Q42: What type of activities should we do in the next 5 years to facilitate the use of new technologies and methodologies in clinical practice?]

In the context of further developing sensorimotor assessments, Neuroscientists believe that paradigms from movement neuroscience can provide more insights than standard clinical assessments (score of 4.2 on a scale of 1 to 5; Q27). Explaining their response further (Q28), they believed that:

1. Tools and methods of neuroscience would provide more precise and objective information about a patient’s movement quality and quantity, which are not available through standard clinical assessments;
2. The use of computational models to better understand recovery (at different levels) would be a useful feature in neurorehabilitation.

In general, they recognized that not all approaches from Neuroscience might be practically feasible for routine clinical use.

### Open comments

The final questions of the survey asked for specific comments for the panel discussion at the ICORR workshop (Q44), and any open comments related to sensorimotor assessments (Q45). A summary of the responses is provided in Table 13.

**Table 13:** Summary of open questions (Q44) and comments (Q45)

Research Engineers	Questions
	• Will insurance companies be willing to reimburse for technology-aided assessment with the current level of standardization and output metrics? • How to combine diverse technologies and the lack of clarity on what exactly to measure? What is the way forward for both these issues?
	Comments
	• It seems that standardization, clinical use, and reimbursement mechanisms are a bit of a chicken and egg problem: standardization requires use, use requires reimbursement, and reimbursement requires standardization.
Clinicians	Questions
	• How to interpret and standardize technical outcome parameters? • Why don’t technology developers collect more information from clinical therapists and patients?
	Comments
	• There is a need for better access to affordable technology for clinical use. • An objective sensory measurement tool would be clinically useful. • Assessment must be reliable, meaningful, simple to administer and learn. • Technology failures cannot occur. We cannot use the time of clinicians and patients by using technically unreliable tools.
Basic Scientists	Questions
	• What is really important to assess for clinicians, therapists, and patients? Are they aligned? • How do researchers developing outcome measures based on neuroscientific principles convince clinicians to trust and use these new tools?
	Comments
	• *“… effects of TBI injuries and stroke are more clearly reflected in movement rather than in imaging, as is improvement due to rehab. It is time to use movement science to do a composite assessment of brain-body and to start building tools which can sense accurately and with reliability.*” • *“There is significant under-estimation of current pressures on clinicians in the public sector. Certain assessments are mandated by Govt. agencies, even though they have limited usefulness clinically. Departments of Health also need to be included in education about technology.”* • There is a need for standardization and widespread implementation.
Medical Industry	Comments
	There are standard requirements of any measurement tool, e.g., validity, reliability, sensitivity to change, inter and intra-rater reliability, training. It is important that these are evaluated in a standardized way.
Others	Questions
	What is the best way to share current research outcomes for clinical therapists to be up-to-date on the latest findings? Such sharing of knowledge can advocate the gradual implementation of technology in clinical activities at different organizational levels.
Patients	Comments
	*“It is important to consider how much time an assessment should take. This time is taken from the rehab time!”*

## Discussion

This work aimed at identifying the barriers and facilitators for technology-driven assessments for sensorimotor function, as perceived by different stakeholder groups. The survey was carried out via an online questionnaire to obtain a broad overview of different viewpoints from across the globe. Our results reflect the opinion of 140 respondents from 23 countries from the Engineer, Clinician, Neuroscientist, Patient and Medical Industry stakeholder groups. There were disparate views on the current bottlenecks to technology-aided sensorimotor assessments, due to differences in stakeholders’ professional backgrounds, their goals, and the primary focus of their work. Nevertheless, we found that stakeholders generally agreed on the value of technology-aided sensorimotor assessments and how to move towards clinical practice.

### Current barriers

An obvious barrier to the adoption of technology-aided assessments could be the lack of demand for such tools, i.e., if assessments are not routinely performed. Previous studies [6, 8, 9] reported low implementation rates of assessments in routine clinical practice, which is consistent with the beliefs of Research Engineers, Basic Scientists and Medical Industry professionals in our questionnaire (section 3.2.1). However, all clinicians reported that they use standard clinical assessments, with approximately 75% using them on a regular basis (50% at least once a week and 24% at least once a month; Table 4). It is possible that the inconsistency between previous studies and our results on clinicians’ reported use of assessments reflects a biased view from our respondents, who may be more inclined to use assessments. But it is also possible that assessments are currently performed more regularly than in the past (based on literature references from 5 to 9 years before our survey). Furthermore, Medical Industry professionals reported that there is a strong interest among their clients in tools for sensorimotor assessments, foreseeing an increase in demand in the coming years. Medical Industry professionals also reported that they invest resources in the development of such tools, and that assessment capabilities are a selling point for their devices. Thus, the lack of need is likely not the reason for the poor adoption of these tools in clinical practice.

It is possible that the available technology does not meet the user needs for sensorimotor assessments due to differences in understanding of the role of assessments in clinical practice among the different stakeholder groups (especially between developers and users). However, we found general agreement across stakeholders. There was agreement among stakeholders that individual reasons were the most important reason for performing assessments (Table 6). Stakeholders also agreed, in general, about the level of detail required from assessments for different purposes (Table 7): communication with government bodies, insurance companies and patients were perceived to require the lowest amount of detail, followed by evaluation or selection of therapies, and finally determining medications or invasive interventions. These results indicate that there is an alignment across stakeholder groups about the general reasons for doing sensorimotor assessments, and the level of detail required for different goals.

Another barrier for adoption of technology-aided assessments could be the lack of access and exposure to available tools. We did not find this to be the case among the respondents in this study; the majority of stakeholders had used or at least observed technology-aided assessments being used (Fig. 5). In particular, around 50% of Clinicians worked with technological tools on a regular basis (at least once a month), and 60–80% of Clinicians used technological tools to measure a wide range of variables relevant to neurorehabilitation (Fig. 4). Although these percentages are not sufficient to suggest widespread adoption of technologies, they are encouraging as they indicate that the main user group of assessment technologies has at least been exposed to such tools. Thus, in a population with access and exposure to neurorehabilitation technology, there were other barriers that were preventing routine use of technology-aided assessments.

The survey identified that practical considerations (e.g., assessment duration, cost, etc.) were some of the main factors hindering clinical use of standard assessments (Table 10). This is in line with Jette, et al. [8], where therapists identified: (a) duration for patients to complete assessments, (b) duration for clinicians to analyze data, and (c) difficulty for patients in completing assessments independently, as the three main barriers for routine assessments. We also found this for technology-aided assessments, where, across stakeholders, lack of time to perform assessments and high cost of technologies were the top two hindering factors (Table 11). Interestingly, Medical Industry professionals chose the lack of reimbursement for time spent in assessments as the second most hindering factor. This highlights the importance of financial aspects that directly impact delivery of care, adding to the cost associated with technology-aided assessments.

Unlike other stakeholders, Research Engineers scored the lack of understanding on how to interpret and use assessment results, and the lack of standardization/ device-dependence of assessment results as the most important factors hindering technology-aided assessments. These highlight the differences in the roles of stakeholder groups: Research Engineers, who are primarily tool developers (Fig. 5a), are aware of the limitations of these devices and the applicability of the different methods used in quantitative assessments. However, this aspect seems to be lost to other (user) stakeholders, which can easily lead to inappropriate use of the developed tools, e.g., application of measures in conditions where they are not valid, comparison of metrics with same name but that are not calculated in a comparable manner, etc. Thus, deeper interdisciplinary interactions need to be fostered to decrease the potential of overlooking stakeholder-specific factors.

In general, Clinicians showed a strong preference for standard tools for carrying out assessments in their work (Table 5). Despite this, Clinicians scored the lack of standards and interpretability of results below the financial burden of technology-aided assessments, indicating that the cost of existing technology was a more pressing problem than the lack of standards. Across stakeholders, the poor understanding of using technology-aided assessments for clinical decision-making, and the lack of standards were rated the third and fourth important factors hindering clinical acceptance. The lack of standards is a known problem with technology-aided assessments, as there is no general consensus on what features of sensorimotor behavior to measure and how, especially in the context of neurorehabilitation. Differences in technologies (e.g., robots vs. sensor-based systems) affect the nature of human-machine interaction between the different devices. This further exacerbates the problem of standardization and could partly explain the current lack of standards given the wide landscape of available technological tools. The tendency is that technology will continue to improve at a fast pace, and this is one reason why it is critical to select a solid base to best explore technological developments rather than re-evaluate every new device.

The issues of standardization, the associated time and cost to perform assessments, and reimbursement for clinical use of technology-aided assessment are intermingled. The circular nature (“chicken-and-egg” problem) of these issues was also raised in the open comments (Table 13): reimbursement requires standardization, standardization requires clinical use, and clinical use requires reimbursement/standardization. These issues are unlikely to be resolved immediately, and require high-quality evidence in favor of technology-aided assessments to demonstrate the value added by these procedures to clinical practice. In their current state, technology-aided assessments are likely not adding much value to clinical practice, and have poor benefit-to-cost (money and time) ratio. In contrast, technologies such as MRI are routinely used given the value they add to clinical practice, and there is little discussion around whether or not to use these procedures, even though they are many times more expensive than most technologies related to sensorimotor assessments. Thus, clearly showing the added value of technology-aided assessments to clinical practice, in terms of improved and efficient assessment, and better clinical decision-making and outcomes, is critical to support their future adoption.

### Suggestions for the future of technology-aided assessments

There was general agreement between the different stakeholders about the activities to be pursued for the next 5 years (Table 12), with standardization of technology-aided assessments ranked highest. This was followed by activities to: (a) understand the link between the different levels of assessment, which is important for therapy planning and clinical decision making; and (b) make routine assessment reimbursable and economically viable. The least ranked activities were cross-disciplinary education, and the development of taxonomies for the field.

The suggestion to focus on standardization of technology-aided assessments is a logical one, but a difficult issue to address. Even without technology, developing standards in sensorimotor assessments requires a number of inter-related choices: the tasks performed and the assessment protocol, tools used for measurements, and the methods used for analysis and interpretation of the results. Technology in this context specifically refers to the tools used for assessment, and it directly influences the tasks that can be performed, the physical variables that can be measured, and the interpretation of results.

In the last decades, robotics has emerged as a viable and safe tool to complement traditional therapy (see [10–12]) and it is a popular technology used by different stakeholders (Fig. 4). These robotic developments, primarily designed for delivering therapy, have also been programmed to measure a great variety of parameters related to sensorimotor function (e.g., [3, 13, 14]) and they have been suggested as standard tools for sensorimotor assessments. Although an appealing idea, using therapeutic robots for assessment is limited because each outcome measure is influenced by robot-specific factors such as the topology of the robot’s kinematic chain, control modalities, etc., which confound the results obtained from these tools. Nevertheless, robotic devices have the potential to become standards in assessments, but they must be specifically designed for the purpose of assessment, e.g., robotic dynamometers or ergometers.

Another way forward is by using technology that has the potential for quick and widespread use with minimal interference to human movements, to promote focused and quicker convergence to a common set of measures/standards. One such example is inertial measurement technology, whose popularity is increasing and is likely to be ubiquitous in the near future. Inertial measurement technology has been used to quantify many existing assessments [15] and movement behaviour in natural (non-laboratory) conditions [16–18]. Thus, this technology could help establish standards in technology-based sensorimotor assessments. However, this technology also has its limitations. For instance, there is currently no standard algorithm for extracting movement kinematics from these devices; several different algorithms for activity counts or steps [19], energy expenditure [20, 21], smoothness [22, 23], etc., exist but were validated in different populations (e.g., walking detection algorithms that work very well in the unimpaired population may not work well with impaired users [24]). This is not ideal for establishing a standard, but it is likely that coming years will see increased work with this particular technology, which hopefully will result in accurate, robust and sensitive methods for quantifying movements. To accelerate and facilitate this, it is relevant to encourage the community to openly share their algorithms and data. Standardization is unlikely to happen if every research group develops their own version of different measures; as highlighted by Ince et al. [25], non-availability of the original software code is a serious impediment to reproducibility – even if algorithms are perfectly described in the literature.

Moreover, there currently exist no standards for accurate and robust data collection with these devices in both laboratory/clinic and real-life settings. In the laboratory/clinic setting, there is a need to standardize tasks, so comparisons can be made between studies. In the real-life setting, there is still a big challenge in interpreting data from these devices operating in unstructured environments and without contextual information. Nevertheless, interesting and potentially useful methods have started surfacing in recent years, e.g., [17, 26], and hopefully these will be extended further in the coming years.

Activities towards standardization can also help promote reimbursement of assessment procedures used in clinical practice, which can address issues with the cost associated with technology-based assessments. Apart from agreeing on a common set of tools to be used for sensorimotor assessments, another important issue that needs to be addressed for developing standards is the development of suitable language and taxonomies for the field [27]. This is essential to ensure there is a common understanding of important constructs in the field among the different stakeholders. Such language is also crucial for formalizing procedures, e.g., [28], and the development of appropriate measures for quantifying different sensorimotor constructs relevant for research and clinical purposes. There have been recent international efforts towards standardizing outcome measures for sensorimotor assessments with and without technology, e.g., [29, 30]. An encouraging thought is that the difficulties currently faced in standardization of technology-aided assessments are not very unlike problems encountered in other fields before standards were established (e.g., [31–33]), or at least openly discussed (e.g., [34]). It would be essential to continue our technical and clinical work in the different fronts, while being aware of how our activities fit in the bigger picture of a standard for technology-aided assessments.

Cross-stakeholder communications would also be essential in this process, and a common language will also make it easy to implement cross-disciplinary education among the different stakeholders. We found surprising that, while different respondents reported having had many interdisciplinary interactions (Table 3), many technology developers had not used nor seen technology for assessments being used (Fig. 5). This could be reflected as a lack of proper understanding of user needs when developing technology, which could lead to devices not being used, and highlight the importance of fostering quality cross-disciplinary education and interactions among stakeholders.

### Limitations of the present work

The results of this survey should be taken in light of some of its shortcomings:

1. An online survey was adopted at the cost of rigor in the data collection process to obtain a broad overview of the different viewpoints from across the globe. This could have resulted in a biased view of the field.
2. The number of responses per stakeholder group was low with large differences in the numbers among the different stakeholders, e.g., there were more than 40 Engineers, but only 3 Patients.
3. The majority of respondents were from developed countries, likely with more access to technologies. Also, given how the survey was advertised, the respondents are likely to be from technology-oriented institutions or institutions already inclined to promote the use of technology. Thus, the views presented are likely a biased view of stakeholders with an interest in the use of technology-driven assessments.
4. To define ‘standard clinical assessments’ and ‘technology-aided assessments’ we relied on picture examples with no written definition. This was subjective and their meanings could have been interpreted differently by different people, affecting some of the responses.
5. Another limitation towards reaching a more representative population was language. The survey was only available in English and we heard from many therapists that their colleagues could not respond to the questionnaire due to insufficient mastery of English. This decreased participation, and could have also led to misinterpretation of questions and/or answers among the respondents.
6. Finally, we did not get any response from policy makers or insurance companies, which are important to understand how the use of technology-aided assessments is promoted in everyday practice [35].

## Conclusions

The current study presented results from an online survey to understand the perceptions of the different stakeholders about the *status quo* of technology-based sensorimotor assessments, in particular: (a) the barriers to their adoption in clinical practice; and (b) the potential avenues for future work to promote use of technology-aided assessments in research and clinical practice. There was general agreement between the stakeholders on the different issues, with some differences between the stakeholders based on their educational and professional backgrounds. Lack of time, high cost and difficulties in interpretability of results from technology-aided sensorimotor assessments were identified as the three major bottlenecks. Standardization of technology-aided assessment was identified as the most important activity to pursue to promote the field. We strongly believe that with continued technical and clinical efforts, and communication between the different stakeholder groups, a suitable standard can be established in the due course of time.

## Additional files

Additional file 1: Responses. (XLSX 49 kb)

Additional file 2: Questionnaire. (PDF 2623 kb)

## Authors’ information

Camila Shirota is a Senior Research Associate at the Rehabilitation Engineering Lab of ETH Zurich, Switzerland. She received her Ph.D. in Biomedical Engineering from Northwestern University for her work at the Rehabilitation Institute of Chicago, in tripping recovery of unilateral transfemoral amputees. She is interested in locomotor control, balance, and rehabilitation.

Sivakumar Balasubramanian is an Associate Professor in Bioengineering at the Christian Medical College, Vellore India. He received his Ph.D. in Bioengineering from Arizona State University, USA. Following this he worked as a research associate in the Human Robotics Group at the Imperial College London, and subsequently worked as a researcher at Tecnalia Research & Innovation (Spain). He is interested in the development and clinical validation of neurorehabilitation technology, and quantitative sensorimotor assessment of neurological conditions.

Alejandro Melendez-Calderon is a Senior Research Scientist in Neuromechanics at the cereneo Advanced Rehabilitation Institute (Switzerland), Guest Researcher at ETH Zurich, and Adjunct Assistant Professor at Northwestern University (USA). He was previously a postdoctoral research fellow at the Rehabilitation Institute of Chicago, and Project Manager and (co-) leader of the Core Technologies Assessments & Reporting at Hocoma AG. He received his doctoral degree from Imperial College London for research in robotics, rehabilitation and human motor control. He is interested in understanding principled mechanisms of human behavior, in particular related to movement control/learning and physical interaction.
