# Supplementary material for: Technology-aided assessments of sensorimotor function: current use, barriers and future directions in the view of different stakeholders
Source: J Neuroeng Rehabil. 2019 Apr 29;16:53. doi: 10.1186/s12984-019-0519-7 (PMC6489331; doi:10.1186/s12984-019-0519-7)
Supplement: Supplementary file 2 — Questionnaire. (PDF 2623 kb) [file 12984_2019_519_MOESM2_ESM.pdf]

# RehabWeek 2017 - Survey on sensorimotor assessments

This is a fully anonymized questionnaire and your email will only be collected if you provide it at the end of the survey.

* Required

## We need your opinion on how to advance technology-aided assessments

It is often said that "technology-aided assessments can provide more objective, sensitive and reliable measurements than traditional clinical scales". However, a significant amount of resources (time, money, man power) is often spent in developing tools that are rarely used in everyday clinical practice.

This survey is intended for all stakeholders in the field of neurorehabilitation (clinicians, therapists, engineers, manufacturers, patients, etc.), to help the community understand each point of view, align efforts, and close the gap between research and everyday clinical practice - specifically in regards to technology-aided sensorimotor assessments.

Thank you for participating in this survey!

After RehabWeek 2017, we will publish the results of this survey. We hope you find this valuable to guide your efforts!

## Get familiar with our definitions

The following image illustrates what we mean by 'standard clinical scales' and 'technology-aided assessments'.

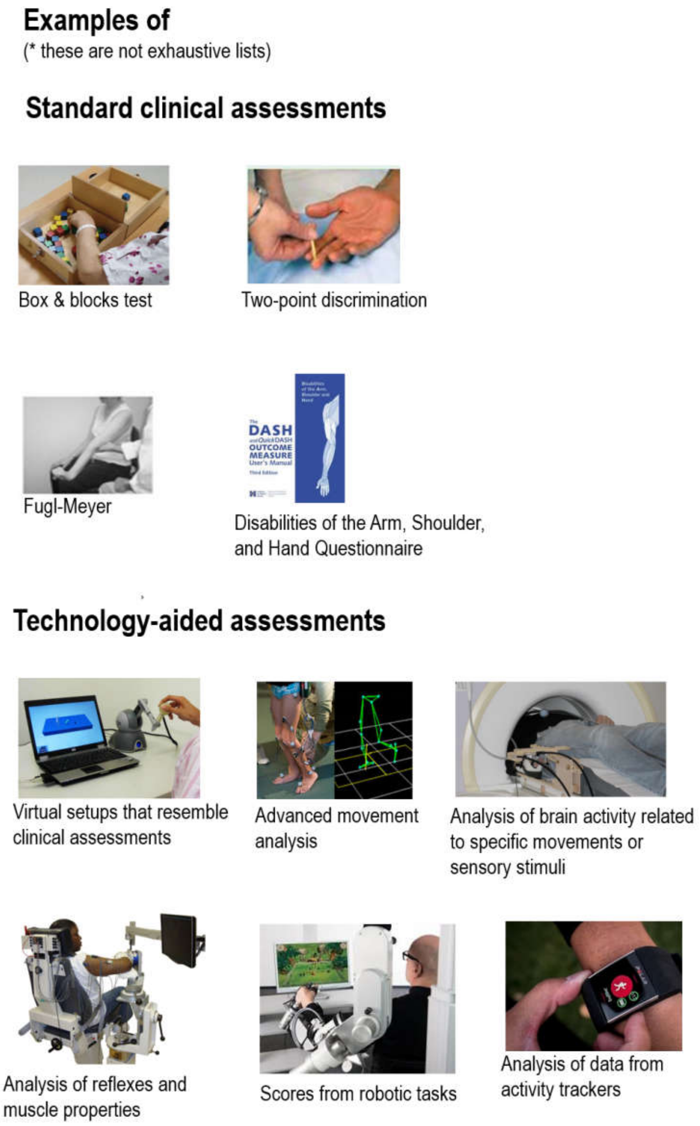


## Tell us about you

1. **What is your gender?** *

*Mark only one oval.*

- ( ) Male
- ( ) Female
- ( ) Prefer not to say

2. **What country do you live/work in?** *

*Mark only one oval.*

- Afghanistan
- Albania
- Algeria
- Andorra
- Angola
- Antigua and Barbuda
- Argentina
- Armenia
- Aruba
- Australia
- Austria
- Azerbaijan
- Bahamas, The
- Bahrain
- Bangladesh
- Barbados
- Belarus
- Belgium
- Belize
- Benin
- Bhutan
- Bolivia
- Bosnia and Herzegovina
- Botswana
- Brazil
- Brunei
- Bulgaria
- Burkina Faso
- Burma
- Burundi
- Cambodia
- Cameroon
- Canada
- Cabo Verde
- Central African Republic
- Chad
- Chile
- China
- Colombia
- Comoros
- Congo, Democratic Republic of the
- Congo, Republic of the
- Costa Rica
- Cote d'Ivoire
- Croatia
- Cuba
- Curacao
- Cyprus
- Czechia
- Denmark
- Djibouti
- Dominica
- Dominican Republic
- East Timor
- Ecuador

- Egypt
- El Salvador
- Equatorial Guinea
- Eritrea
- Estonia
- Ethiopia
- Fiji
- Finland
- France
- Gabon
- Gambia, The
- Georgia
- Germany
- Ghana
- Greece
- Grenada
- Guatemala
- Guinea
- Guinea-Bissau
- Guyana
- Haiti
- Holy See
- Honduras
- Hong Kong
- Hungary
- Iceland
- India
- Indonesia
- Iran
- Iraq
- Ireland
- Israel
- Italy
- Jamaica
- Japan
- Jordan
- Kazakhstan
- Kenya
- Kiribati
- Korea, North
- Korea, South
- Kosovo
- Kuwait
- Kyrgyzstan
- Laos
- Latvia
- Lebanon
- Lesotho
- Liberia
- Libya
- Liechtenstein
- Lithuania
- Luxembourg
- Macau
- Macedonia
- Madagascar
- Malawi

- Malaysia
- Maldives
- Mali
- Malta
- Marshall Islands
- Mauritania
- Mauritius
- Mexico
- Micronesia
- Moldova
- Monaco
- Mongolia
- Montenegro
- Morocco
- Mozambique
- Namibia
- Nauru
- Nepal
- Netherlands
- New Zealand
- Nicaragua
- Niger
- Nigeria
- North Korea
- Norway
- Oman
- Pakistan
- Palau
- Palestinian Territories
- Panama
- Papua New Guinea
- Paraguay
- Peru
- Philippines
- Poland
- Portugal
- Qatar
- Romania
- Russia
- Rwanda
- Saint Kitts and Nevis
- Saint Lucia
- Saint Vincent and the Grenadines
- Samoa
- San Marino
- Sao Tome and Principe
- Saudi Arabia
- Senegal
- Serbia
- Seychelles
- Sierra Leone
- Singapore
- Sint Maarten
- Slovakia
- Slovenia
- Solomon Islands
- Somalia

- South Africa
- South Korea
- South Sudan
- Spain
- Sri Lanka
- Sudan
- Suriname
- Swaziland
- Sweden
- Switzerland
- Syria
- Taiwan
- Tajikistan
- Tanzania
- Thailand
- Timor-Leste
- Togo
- Tonga
- Trinidad and Tobago
- Tunisia
- Turkey
- Turkmenistan
- Tuvalu
- Uganda
- Ukraine
- United Arab Emirates
- United Kingdom
- Uruguay
- Uzbekistan
- Vanuatu
- Venezuela
- Vietnam
- Yemen
- Zambia
- Zimbabwe

3. **Do you currently receive therapy due to a neuromuscular disorder?** *

*Mark only one oval.*

- Yes *Skip to question 17.*
- No *Skip to question 4.*

## Tell us about you

4. **How many years of professional experience do you have in the field of rehabilitation?*** *

* Including: clinical work, research, technology development, sales, policy making, etc.

*Mark only one oval.*

- < 1 Year
- 1 to 5 Years
- 6 to 10 Years
- > 10 Years

5. **What kind of exposure have you had with technology-aided assessments?** *

*Check all that apply.*

- [ ] I have developed technology
- [ ] I have used technology
- [ ] I have observed technology being used
- [ ] I have no experience
- [ ] Other: ____________________

6. **What is your profession?** *

If you have more than one profession, select the one you most identify with. If you are a student, select your field of study.

*Mark only one oval.*

- Therapist (research-focused) *Skip to question 7.*
- Therapist (clinic-focused) *Skip to question 7.*
- Medical doctor *Skip to question 7.*
- Hospital / clinic administrator *Skip to question 29.*
- Engineer (academic or non-profit institution research) *Skip to question 13.*
- Engineer (medical industry) *Skip to question 13.*
- Neuroscientist *Skip to question 24.*
- Manager (medical industry) *Skip to question 32.*
- Other (medical industry) *Skip to question 32.*
- Sales representative of medical technologies *Skip to question 32.*
- Policy maker (e.g., work for a goverment organization) *Skip to question 37.*
- Insurance representative *Skip to question 37.*
- Other: ______________________ *Skip to question 37.*

## Tell us about your experience [Clinician]

7. **What kind of unit do you work in?** *

If you work in more than one, select the place where you spend most of your time.

*Mark only one oval.*

- Acute-care unit
- Inpatient unit ("small", < 50 beds)
- Inpatient unit ("large", > 50 beds)
- Outpatient unit ("small", < 50 patients)
- Outpatient unit ("large", > 50 patients)
- Skilled nursing facility
- Home care
- Other: ______________________

8. **How often are these technologies used in your work (not restricted to assessments)?** *

*Mark only one oval per row.*

| | Never | Very rarely / only in select cases | At least once a month | At least once a week | Everyday |
|---|---|---|---|---|---|
| Robotic devices (i.e., with motors) | | | | | |
| Motion sensors (e.g., IMUs, activity trackers, electrogoniometers, cameras) | | | | | |
| Devices to measure brain activity (e.g., EEG, MRI scanners) | | | | | |
| Devices to measure muscle activity (e.g., EMG) | | | | | |
| Virtual reality / gaming environments (e.g., Nintendo Wii) | | | | | |
| Force/presure sensors (e.g., dynamometers, force plates, pressure insoles) | | | | | |
| Non-robotic devices with sensors (e.g., ergometers) | | | | | |

9. **How do you assess the sensorimotor ability of your patients?** *

Rank the following options in your order of preference.

*Mark only one oval per row.*

| | 1: Least preferred | 2 | 3 | 4 | 5 | 6 | 7: Most preferred |
|---|---|---|---|---|---|---|---|
| Standard clinical assessments (scales or questionnaires) | | | | | | | |
| Low-tech measurement tools (e.g., goniometer) | | | | | | | |
| Verbal communication | | | | | | | |
| Visual inspection / observation | | | | | | | |
| Using technology | | | | | | | |
| Physical manipulation | | | | | | | |
| Non-standardized tasks or questionnaires | | | | | | | |

10. **How often do you (or your personnel) perform standard clinical assessments on a given patient during routine clinical practice?** *

*Mark only one oval.*

- Never
- Very rarely / only in select cases
- Only during entry and/or discharge
- At least one a month
- At least once a week

11. **How often do you (or your personnel) use any kind of technology to perform assessments on a given patient during routine clinical practice?** *

*Mark only one oval.*

- Never
- Very rarely / only in select cases
- Only during entry and/or discharge
- At least one a month
- At least once a week

12. **In your work, what kind of interactions have you had with technology developers?** *

*Mark only one oval per row.*

| | Never | 1 case | 2-5 different cases | >5 different cases |
|---|---|---|---|---|
| Direct interaction for technology development | | | | |
| Direct interaction for clinical validation of technology-aided assessments | | | | |

*Skip to question 37.*

## Tell us about your experience [Engineer]

13. **How often are these technologies used in your work environment?** *

*Mark only one oval per row.*

| | Never | Very rarely / only in select cases | At least once a month | At least once a week | Everyday |
|---|---|---|---|---|---|
| Devices to measure muscle activity (e.g., EMG) | | | | | |
| Robotic devices (i.e., with motors) | | | | | |
| Non-robotic devices with sensors (e.g., ergometers) | | | | | |
| Force/presure sensors (e.g., dynamometers, force plates, pressure insoles) | | | | | |
| Virtual reality / gaming environments (e.g., Nintendo Wii) | | | | | |
| Devices to measure brain activity (e.g., EEG, MRI scanners) | | | | | |
| Motion sensors (e.g., IMUs, activity trackers, electrogoniometers, cameras) | | | | | |

14. **In your work, what kind of interactions have you had with patients?** *
*Mark only one oval per row.*

| | Never | 1-5 patients | 5-10 patients | >10 patients |
|---|---|---|---|---|
| Observed during therapy sessions | ◯ | ◯ | ◯ | ◯ |
| Direct interaction for technology development | ◯ | ◯ | ◯ | ◯ |
| Direct interaction through clinical studies | ◯ | ◯ | ◯ | ◯ |

15. **In your work, what kind of interactions have you had with therapists?** *
*Mark only one oval per row.*

| | Never | 1-5 therapists | 5-10 therapists | >10 therapists |
|---|---|---|---|---|
| Observed during therapy sessions | ◯ | ◯ | ◯ | ◯ |
| Direct interaction through clinical studies | ◯ | ◯ | ◯ | ◯ |
| Direct interaction for technology development | ◯ | ◯ | ◯ | ◯ |

16. **How often do you think therapists perform standard clinical assessments on a given patient during routine clinical practice?** *
*Mark only one oval.*

- ◯ Never
- ◯ Very rarely / only in select cases
- ◯ Only during entry and/or discharge
- ◯ At least one a month
- ◯ At least once a week

*Skip to question 37.*

## Tell us about your experience [Patient]

17. **In what kind of unit are you being treated?** *
*Mark only one oval.*

- ◯ Acute-care unit
- ◯ Inpatient unit ("small", < 50 beds)
- ◯ Inpatient unit ("large", > 50 beds)
- ◯ Outpatient unit ("small", < 50 patients)
- ◯ Outpatient unit ("large", > 50 patients)
- ◯ Skilled nursing facility
- ◯ Home care
- ◯ Other: ______________________

18. **How often do you do clinical assessments during your therapy sessions?** *
*Mark only one oval.*

- ◯ Never
- ◯ Very rarely /only in select cases
- ◯ Only during entry and/or discharge
- ◯ At least one a month
- ◯ At least once a week

19. **How often do your therapists use technological tools to assess you during therapy sessions?** *
*Mark only one oval.*

- ◯ Never
- ◯ Very rarely / only in select cases
- ◯ Only during entry and/or discharge
- ◯ At least one a month
- ◯ At least once a week

20. **Do you see any value in having your abilities assessed by a professional?**
*Mark only one oval.*

- ◯ Yes
- ◯ No
- ◯ Maybe

21. **Are you made aware of the scores from your clinical assessments?**

*Mark only one oval.*

- Yes
- No
- Sometimes

22. **Do you use any technological tool on your own to assess your progress?** *

*Mark only one oval.*

- Yes *Skip to question 23.*
- No *Skip to question 37.*

## Tell us about your experience [Patient]

23. **Please specify what kind of technologies you use** *

_______________________

*Skip to question 37.*

## Tell us about your experience [Neuroscientist]

24. **In your work, what kind of interactions have you had with patients?** *

*Mark only one oval per row.*

| | Never | 1-5 patients | 5-10 patients | >10 patients |
|---|---|---|---|---|
| Observed during therapy sessions | | | | |
| Direct interaction through neuroscience studies on sensorimotor control | | | | |
| Direct interaction through clinical studies on therapeutic interventions | | | | |

25. **In your work, what kind of interactions have you had with therapists?** *

*Mark only one oval per row.*

| | Never | 1-5 therapists | 5-10 therapists | >10 therapists |
|---|---|---|---|---|
| Observed during therapy sessions | | | | |
| Direct interaction through neuroscience studies on sensorimotor control | | | | |
| Direct interaction through clinical studies on therapeutic interventions | | | | |

26. **How often do you think therapists perform standard clinical assessments on a given patient during routine clinical practice?** *

*Mark only one oval.*

- Never
- Very rarely /only in select cases
- Only during entry and/or discharge
- At least one a month
- At least once a week

27. **Do you think paradigms from movement neuroscience (e.g., force-field adaptation paradigms, bimanual circle drawing, fMRI paradigms, etc.) can provide more insights than standard clinical assessements for assessing sensorimotor impairments in the clinical practice?** *

*Mark only one oval.*

| | 1 | 2 | 3 | 4 | 5 | |
|---|---|---|---|---|---|---|
| Completely disagree | | | | | | Fully agree |

28. **How?** *
Please elaborate on your previous answer.

*Skip to question 37.*

## Tell us about your experience [Clinical Administrator]

29. **How often are these technologies used in your work (not restricted to assessments)?** *
*Mark only one oval per row.*

| | Never | Very rarely / only in select cases | At least once a month | At least once a week | Everyday |
|---|---|---|---|---|---|
| Virtual reality / gaming environments (e.g., Nintendo Wii) | ◯ | ◯ | ◯ | ◯ | ◯ |
| Robotic devices (i.e. with motors) | ◯ | ◯ | ◯ | ◯ | ◯ |
| Force/presure sensors (e.g. dynamometers, force plates, pressure insoles) | ◯ | ◯ | ◯ | ◯ | ◯ |
| Motion sensors (e.g. IMUs, activity trackers, electrogoniometers, cameras) | ◯ | ◯ | ◯ | ◯ | ◯ |
| Devices to measure muscle activity (e.g. EMG) | ◯ | ◯ | ◯ | ◯ | ◯ |
| Devices to measure brain activity (e.g. EEG, MRI scanners) | ◯ | ◯ | ◯ | ◯ | ◯ |
| Non-robotic devices with sensors (e.g. ergometers) | ◯ | ◯ | ◯ | ◯ | ◯ |

30. **How often are these technologies used in your work (for assessments only)?** *
*Mark only one oval per row.*

| | Never | Very rarely / only in select cases | At least once a month | At least once a week | Everyday |
|---|---|---|---|---|---|
| Devices to measure muscle activity (e.g. EMG) | ◯ | ◯ | ◯ | ◯ | ◯ |
| Robotic devices (i.e. with motors) | ◯ | ◯ | ◯ | ◯ | ◯ |
| Devices to measure brain activity (e.g. EEG, MRI scanners) | ◯ | ◯ | ◯ | ◯ | ◯ |
| Motion sensors (e.g. IMUs, activity trackers, electrogoniometers, cameras) | ◯ | ◯ | ◯ | ◯ | ◯ |
| Force/presure sensors (e.g. dynamometers, force plates, pressure insoles) | ◯ | ◯ | ◯ | ◯ | ◯ |
| Virtual reality / gaming environments (e.g., Nintendo Wii) | ◯ | ◯ | ◯ | ◯ | ◯ |
| Non-robotic devices with sensors (e.g. ergometers) | ◯ | ◯ | ◯ | ◯ | ◯ |

31. **How often does your personnel perform standard clinical assessments on a given patient during routine clinical practice?** *
*Mark only one oval.*

- ◯ Never
- ◯ Very rarely /only in select cases
- ◯ Only during entry and/or discharge
- ◯ At least one a month
- ◯ At least once a week

*Skip to question 37.*

## Tell us about your experience [Medical industry]

32. **What kind of technologies do you provide?** *

*Check all that apply.*

- [ ] Motion sensors (e.g., IMUs, activity trackers, electrogoniometers, cameras)
- [ ] Devices to measure muscle activity (e.g., EMG)
- [ ] Devices to measure brain activity (e.g., EEG, MRI scanners)
- [ ] Virtual reality /gaming environments
- [ ] Robotic devices (i.e., with motors)
- [ ] Non-robotic devices with sensors (e.g., ergometers)
- [ ] Force/presure sensors (e.g., dynamometers, force plates, pressure insoles)
- [ ] Other: ____________________

33. **What is the current interest in assessment tools?** *

*Mark only one oval per row.*

| | I do not know | 0: No interest | 1 | 2 | 3 | 4 | 5: Everyone shows interest |
|---|---|---|---|---|---|---|---|
| From regulatory bodies | ◯ | ◯ | ◯ | ◯ | ◯ | ◯ | ◯ |
| From therapists | ◯ | ◯ | ◯ | ◯ | ◯ | ◯ | ◯ |
| From medical doctors | ◯ | ◯ | ◯ | ◯ | ◯ | ◯ | ◯ |
| From hospital administrators | ◯ | ◯ | ◯ | ◯ | ◯ | ◯ | ◯ |
| From insurance companies | ◯ | ◯ | ◯ | ◯ | ◯ | ◯ | ◯ |
| From researchers | ◯ | ◯ | ◯ | ◯ | ◯ | ◯ | ◯ |
| From patients | ◯ | ◯ | ◯ | ◯ | ◯ | ◯ | ◯ |

34. **How much resource (time, money, personnel, etc.) do you allocate on developing (if you are a producer) or on highlighting (if you are a distributor) assessment capabilities of the devices you provide?** *

*Mark only one oval per row.*

| | 0: Zero resources | 1 | 2: Same | 3 | 4: Double | 5: More than double | I only produce / sell assessment devices |
|---|---|---|---|---|---|---|---|
| Relative to resources spent in therapeutic capabilities | ◯ | ◯ | ◯ | ◯ | ◯ | ◯ | ◯ |

35. **How much of a selling point are the assessment capabilities of a device?** *

*Mark only one oval.*

| | 1 | 2 | 3 | 4 | 5 | |
|---|---|---|---|---|---|---|
| Do not make any difference | ◯ | ◯ | ◯ | ◯ | ◯ | Determining factor |

36. **Do you foresee any changes in market demand for assessment tools in the coming years?** *

*Mark only one oval.*

- ◯ Yes, I foresee an INCREASE in demand
- ◯ Yes, I foresee a DECREASE in demand
- ◯ No

## Tell us your opinion [general]

37. **What is a reasonable amount of time for a sensorimotor assessment that has to be performed at least once a week (per patient) - from beginning of setup to end of undoing setup?**

*Mark only one oval.*

- ◯ Less than 1 minute
- ◯ 1-5 minutes
- ◯ 6-15 minutes
- ◯ 16-30 minutes
- ◯ 31-60 minutes
- ◯ More than 60 minutes

38. **What are the main motivators to perform and document results from routine assessments (standard clinical or technology-aided)?** *

While multiple levels may be equally important, please rank the options.

**Individual level**

- Understand function, impairment, etc. of a specific patient
- Individual treatment planning
- Individualized evaluation of treatment / intervention
- Communication among medical team to discuss individual case

**Institutional level**

- Resource planning (e.g. allocation of therapist to different patients)
- Indicators of quality assurance inside the clinic / hospital
- Evaluation of services that are provided by the clinic / hospital
- Evaluation of health care delivery models

**Social level**

- Eligibility criteria for social security benefits, disability pensions, insurance, etc.
- Development of guidelines, legislations, policies
- Understand needs of different persons with various levels of disability

*Mark only one oval per row.*

| | 1: Least important | 2 | 3: Most important |
|---|---|---|---|
| Institutional level reasons | | | |
| Individual level reasons | | | |
| Social level reasons | | | |

39. **In your opinion, which is the minimum level of detail needed in the quantification of sensorimotor problems for the following objectives:** *

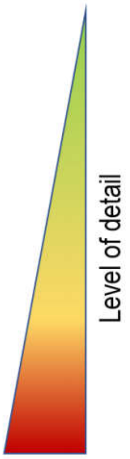


**Activity**
(e.g., walking, eating, dressing, general mobility)

**High-level functions**
(e.g., measures of dexterity, coordination, motor learning ability, balance)

**Low-level functions**
(e.g., measures of strength, reflexes, proprioception)

**Structural / biological**
(e.g., measures of changes in muscle tissue properties, integrity of corticospinal tract)

*Mark only one oval per row.*

| | Activity (lowest level of detail) | High-level functions | Low-level functions | Structural / biological (highest level of detail) |
|---|---|---|---|---|
| To determine medications or invasive interventions (e.g., surgery) | | | | |
| To discuss progress (therapist/clinician with patient) | | | | |
| To determine physical therapy interventions | | | | |
| To monitor progress of patients at home | | | | |
| For patients to monitor their own progress | | | | |
| To report progress to goverment bodies / insurance companies | | | | |
| To discuss patient progress (therapist with clinician) | | | | |
| To gather evidence of effectiveness of interventions | | | | |

40. **How much do issues in each of the following categories hinder the routine use of assessments (standard clinical or technology-aided) in the clinical practice?** *

While multiple levels may be equally important, please rank the options.

**Knowledge, Education, and Perceived Value, e.g.,**

- Lack of knowledge about reliability and validity of different assessment instruments
- Perceived value on the existing assessment instruments
- Lack of trained personnel to perform different assessments

**Support/Priority, e.g.,**

- Support from management / clinic administrators
- Imposition of assessment tools by management
- Inappropriate use of outcome data

**Practical Considerations, e.g.,**

- Time to perform assessments
- Number of assessments required to capture all important aspects
- Lack of funding or excessive costs
- Difficulty of using instruments required for the assessments

**Patient Considerations, e.g.,**

- Information provided by assessments is too subjective and not useful (e.g., to plan therapy interventions)
- Not all patients can complete the assessments (e.g., inability of the patient to understand instructions, complicated tasks)

*Duncan, E.A. and Murray, J., 2012. The barriers and facilitators to routine outcome measurement by allied health professionals in practice: a systematic review. BMC health services research

*Mark only one oval per row.*

| | 1: Least significant factors | 2 | 3 | 4: Most significant factors |
|---|---|---|---|---|
| Patient Considerations | | | | |
| Support/Priority | | | | |
| Knowledge, Education, and Perceived Value | | | | |
| Practical Considerations | | | | |

41. **What are the major bottlenecks in implementing technology-aided sensorimotor assessments in routine clinical practice?** *

Rank in order of importance.

*Mark only one oval per row.*

| | 1: Smallest bottleneck | 2 | 3 | 4 | 5 | 6 | 7 | 8: Biggest bottleneck |
|---|---|---|---|---|---|---|---|---|
| Lack of understanding on how to interpret and use assessment results | | | | | | | | |
| Lack of tools that measure the correct variables | | | | | | | | |
| Technology is not mature enough (e.g., devices break too often) | | | | | | | | |
| Lack of educational programs on how to use technology | | | | | | | | |
| Lack of time to perform assessments | | | | | | | | |
| Results from assessments are highly device-dependent / lack of standardization | | | | | | | | |
| Lack of reimbursement for time spent in assessments | | | | | | | | |
| Technologies are too expensive | | | | | | | | |

42. **What type of activities should we do in the next 5 years to facilitate the use of new technologies and methodologies in clinical practice?** *

Rank in order of importance.
*Mark only one oval per row.*

| | 1: Lowest priority | 2 | 3 | 4 | 5: Highest priority |
|---|---|---|---|---|---|
| Promotion of cross-disciplinary education and communication | ⬭ | ⬭ | ⬭ | ⬭ | ⬭ |
| Better understand the link between different levels of assessment (i.e., activity, high-level function, low-level function, and structural/biological) | ⬭ | ⬭ | ⬭ | ⬭ | ⬭ |
| Proper definition of taxonomies and terms used in the field | ⬭ | ⬭ | ⬭ | ⬭ | ⬭ |
| Standardization of technology-aided assessments (outcome measures and protocols) | ⬭ | ⬭ | ⬭ | ⬭ | ⬭ |
| Make routine assessment procedures reimbursable or economically feasible | ⬭ | ⬭ | ⬭ | ⬭ | ⬭ |

## Final questions

43. **Will you be attending RehabWeek 2017?** *

*Mark only one oval.*

- ⬭ Yes
- ⬭ No

44. **Are there any specific questions you would like us to raise during the expert panel discussion at the workshop "What do we want from technology-aided sensorimotor assessments? –a multi-stakeholder discussion" at RehabWeek 2017?**

________________________________

________________________________

________________________________

________________________________

________________________________

45. **Do you have any further comments?**

________________________________

________________________________

________________________________

________________________________

________________________________

46. **If you would like to be informed of the results of the survey, please enter your email below.**

We only collect your email address if you enter it below.

________________________________

Powered by
Google Forms
